# Anti-Inflammatory Effects of Melatonin in Rats with Induced Type 2 Diabetes Mellitus

**DOI:** 10.3390/life12040574

**Published:** 2022-04-12

**Authors:** Hande Yapislar, Ebru Haciosmanoglu, Turkan Sarioglu, Sevgin Degirmencioglu, Ibrahim Sogut, Michael Poteser, Cem Ekmekcioglu

**Affiliations:** 1 Department of Physiology, Faculty of Medicine, Acibadem Mehmet Ali Aydinlar University, 34684 Istanbul, Turkey; 2 Department of Biophysics, Faculty of Medicine, Bezmialem Vakif University, 34093 Istanbul, Turkey; ehaciosmanoglu@bezmialem.edu.tr; 3 Department of Histology and Embryology, Fundamental Sciences, Faculty of Dentistry, Istanbul Kent University, 34433 Istanbul, Turkey; turkan.sarioglu@kent.edu.tr; 4 Department of Biochemistry, Faculty of Medicine, Istanbul Arel University, 34010 Istanbul, Turkey; sevgindegirmencioglu@arel.edu.tr; 5 Department of Biochemistry, Faculty of Medicine, Demiroglu Bilim University, 34394 Istanbul, Turkey; ibrahim.sogut@demiroglu.bilim.edu.tr; 6 Department of Environmental Health, Center for Public Health, Medical University of Vienna, 1090 Vienna, Austria; michael.poteser@meduniwien.ac.at (M.P.); cem.ekmekcioglu@meduniwien.ac.at (C.E.)

**Keywords:** type 2 diabetes mellitus, experimental diabetes mellitus, melatonin, MT2, inflammation

## Abstract

Introduction: Insulin resistance is associated with a pro-inflammatory state increasing the risk for complications in patients with type 2 diabetes mellitus (T2DM). In addition to its chronobiotic effects, the pineal hormone melatonin is known to exert anti-inflammatory and antioxidant effects. Melatonin was also suggested to affect insulin secretion. The aim of this study was therefore to investigate the effect of melatonin on inflammation in diabetic rats and to study the possible involvement of the melatonin receptor, MT2. Materials and Methods: Male Sprague Dawley rats were randomly divided into four experimental groups (*n* = 10 per group): (1) control, (2) streptozotocin/nicotinamide induced diabetes type 2 (T2DM), (3) T2DM treated with melatonin (500 µg/kg/day), and (4) T2DM treated with melatonin (500 µg/kg/day for 6 weeks) and the selective MT2 receptor antagonist luzindole (0.25 g/kg/day for 6 weeks). Blood samples were taken for biochemical parameters and various tissue samples (liver, adipose tissue, brain) were removed for immunohistochemistry (IHC), Western blot (WB), and Q-PCR analyses, respectively. Results: Melatonin significantly reduced increased blood levels of liver transaminases (AST, ALT), blood urea nitrogen (BUN), triglyceride, very low-density lipoprotein (VLDL), and cholesterol in diabetic rats with luzindole treatment partly reversing this effect regarding the lipids. Furthermore, the liver and adipose tissues of T2DM rats treated with melatonin showed lower expression of the inflammatory markers IL-1β, IL-6, TNF-α, and NF-κB as compared to the T2DM group without melatonin. The results also showed that the MT2 receptor is at least partly involved in the protective effects of melatonin. Conclusions: Our results suggest that melatonin exerts relevant anti-inflammatory effects on various tissues in type 2 diabetic rats.

## 1. Introduction

The worldwide prevalence of diabetes mellitus (DM) is increasing at an alarming rate. According to data from the World Health Organization, diabetes will be the seventh leading cause of death in 2030 [1]. Among DM types, type 2 diabetes mellitus (T2DM) accounts for more than 90% of DM cases and is characterized by hyperglycemia, insulin resistance in target tissues along with several comorbidities, including obesity, cardiovascular risks, renal failure, and retinopathy [2].

Insulin resistance is associated with a pro-inflammatory state increasing the risk for complications in T2DM [2,3]. In this regard, several studies in patients with T2DM have reported higher secretion rates of inflammatory mediators such as IL-1β, IL-6, TNF-α and their connection to complications and beta-cell disorders [2,4]. The major role of IL-1β and TNF-α in particular insulin resistance has been shown repeatedly. TNF-α impairs insulin signaling and decreases the expression of the insulin receptor [5]. IL-1β, a key mediator of the inflammatory response, is also adversely involved in blood glucose control and beta-cell dysfunction [6]. For example, a previous study in patients with T2DM indicated that the blockade of the interleukin-1 receptor improves glycemic control through enhanced beta-cell secretory function [7].

Melatonin (N-acetyl-5-methoxytryptamine) is a circulating hormone that is mainly synthesized and released by the pineal gland. Its secretion is coupled to a robust circadian rhythm with the highest blood levels observed at night between approximately 2 and 4 a.m. [8]. In addition to its chronobiotic and sleep-inducing properties, melatonin is also well known for its antioxidant and anti-inflammatory effects [9,10], and several studies in the scientific literature have demonstrated the anti-inflammatory effects of melatonin in different disease models and conditions [11,12,13,14]. In this regard, both clinical and experimental data have provided evidence that melatonin reduces the secretion of pro-inflammatory cytokines and adhesion molecules and modulates inflammatory parameters [9]. In addition, it was shown that melatonin exerted protective effects on inflammation related to aging [15] and also alcoholic injury in the liver [16]. Furthermore, melatonin reduced inflammation in the central nervous system, which is suggested to be related to several neurological diseases [9], and also exerted an analgesic effect in inflammatory pain conditions [17].

In addition to its anti-inflammatory effects, melatonin was also found to be associated with insulin secretion and DM [18]. In this regard, studies suggest a direct link between insulin secretion from beta cells, glucose levels, and melatonin secretion [19,20]. For example, a negative correlation between the nocturnal melatonin peak and insulin drop levels in serum was described [21,22]. Moreover, in a study by Peschke et al., it was reported that patients with type 2 diabetes show lower circulating melatonin levels and higher insulin levels, respectively, with a statistically significant inverse correlation between these two hormones [23].

Melatonin mainly exerts its effects after binding to two melatonin receptors (MT), MT1 and MT2, respectively [24]. Both receptors have different expression ratios in various tissues [24,25]. Previous studies suggest that the immunomodulatory functions of melatonin are at least partly mediated after binding to the MT2 receptor [24,25,26,27]. Different expression profiles of both types of receptors and signal transduction pathways have been identified in various tissues and cell types [24]. Melatonin receptors belong to the G-protein coupled receptors (GPCR) and are thus part of the cAMP-pathway. Dissociation of Gi stimulates the activity of PLC (phospholipase C) that converts PIP2 (phosphatidylinositol phosphate) into DAG (diacylglycerol) and IP3 (inositol trisphosphate), both elements inducing intracellular calcium signals [28].

The aim of this study was to investigate the effect of melatonin on constitutively enhanced inflammation in diabetic rats and to also study the possible involvement of the MT2 receptor. Another aim was to analyze the liver and metabolic blood profiles of melatonin-treated rats since previous studies showed beneficial effects of melatonin in rodents with diabetes or metabolic challenges [29,30,31,32,33].

## 2. Materials and Methods

### 2.1. Animals and Experimental Protocol

All experiments on animals were reviewed and approved by the TUBITAK (HADYEK) Ethical Committee (Approval No: 16563500-111-190) in Turkey. Male Sprague Dawley rats were purchased from TUBITAK MAM and were used for the experiments. After rats reached 200 g body weight T2DM was induced by intraperitoneal (i.p.) injection of 100 mg/kg NAD (nicotinamide adenine dinucleotide) and 50 mg/kg STZ (streptozotocin) respectively. STZ was applied 15 min after NAD administration. Forty-eight hours later, blood glucose levels were measured by a glucometer and rats with glucose levels of ≥250 mg/dL were included in the study.

Rats were randomly allocated to the following four experimental groups (*n* = 10 per group): (1) control, (2) streptozotocin/nicotinamide treated (T2DM), (3) T2DM treated with melatonin (500 µg/kg/day), and (4) T2DM treated with melatonin (500 µg/kg/day) and the selective MT2 receptor antagonist luzindole (0.25 g/kg/day). Melatonin and luzindole treatments were started 48 h after the induction of diabetes with NAD + STZ. Both melatonin and luzindole were administered by daily i.p. injections for 6 weeks. During the treatment body weight was measured once a week. All animals survived during the experiment and the prosperity of none of them decreased according to ethical welfare. At the end of the 6 weeks, animals were fasted overnight, anesthetized using Ketamin/Rompun (50/10 mg/kg i.p.), and sacrificed by cervical dislocation. Blood samples (5 mL) were taken by their jugular veins and collected in tubes containing heparin to subsequently analyze biochemical parameters. Tissue samples were removed and weighed. One-half of the tissue was immersed in paraformaldehyde solution for immunohistochemistry (IHC) applications and the other half was frozen quickly in liquid nitrogen and stored at −80 °C for Western blot (WB) and Q-PCR analyses.

### 2.2. Measurement of Biochemical Parameters

Blood samples were centrifuged at 2500× *g* for 10 min at 4 °C and serum was separated afterwards. Serum alanine and aspartate aminotransferases (ALT, AST) and blood urea nitrogen (BUN), triglyceride, cholesterol, and very-low-density lipoprotein (VLDL) levels were analyzed in serum on a Roche-HITACHI Cobas c311 auto analyzer (Roche Molecular Systems, Branchburg, NJ, USA) by using commercial Roche kits.

### 2.3. Immunohistochemistry (IHC) Protocol

For histopathologic evaluation, routine paraffin wax embedding procedures were applied. Following fixation, tissues were dehydrated in graded ethanol series, clarification process was completed in xylene and slides embedded in paraffin. Then, 5 μm-thick slices were cut via a microtome (Leica RM2235). Formalin-fixed and paraffin-embedded tissue samples were further processed for the evaluation of the severity of tissue inflammation.

After incubation at 56 °C for 12 h, sections were deparaffinized in xylene and the rehydration process was applied through a descending series of alcohol to water. Antigen retrieval was performed by incubation of the sample in 10% citrate buffer (pH 6.0) at 250 °C for 6 min, with subsequent cooling to room temperature for 30 min. Then, tissue sections on slides were marked with a hydrophobic pen and rinsed in phosphate-buffered saline (PBS) with 5% Tween. For protein blocking and non-specific binding, the sections were incubated in 3% H_2_O_2_ for 20 min in dark. After rinsing in phosphate-buffered saline (PBS) the Anti-Polyvalent HRP Kit (Invitrogen, Waltham, MA, USA) was used for the following steps. To reduce non-specific staining, sections were pretreated with the blocking solution for 20 min in a humidity chamber. After removing the blocking solution, slides were covered with primary antibodies used against IL-1β (Santa Cruz, CA, USA), IL-6 (Abnova, Taiwan), TNF-α (Novus Biologicals, Centennial, CO, USA), and NF-κB (Santa-Cruz, CA, USA). Primary antibodies were directly applied on the sections and the slides were incubated overnight at 4 °C in a humidified chamber. The negative control was incubated with a blocking solution without the primary antibody. After washing (3 × 5 min) in PBS-Tween, sections were incubated with HRP-Streptavidin for 20 min in a humidified chamber and washed with PBS-Tween (3 × 5 min). Freshly prepared AEC (Aminoethyl Carbazole, Invitrogen, Waltham, MA, USA) was applied as a chromogen for 8–15 min at room temperature. After the reaction was stopped by washing with deionized water, sections were counterstained with hematoxylin and washed under dripping water for 10–15 min for developing purple color. Finally, sections were covered with a fixative, aqueous mounting solution (Bio-optica), and the stained sections were examined for IL-1β, IL-6, TNF-α, and NF-κB with a BX53 Olympus Camera (DP72 Olympus Software, Ver. 5174). Immunoreactivity for the reference inflammation markers was scored by counting the number of positively stained cell nuclei and expressing this as a percentage of the total number of cell nuclei counted.

### 2.4. Protein Extraction Protocol from Tissues

Tissue samples were homogenized with a homogenizer in RIPA lysis buffer containing a protease inhibitor cocktail (Santa Cruz-sc-24948, Santa Cruz, CA, USA). Homogenates were centrifuged at 13,000 rpm for 10 min, and supernatants were obtained and kept at −80 °C until subsequent analyses. Protein concentrations were determined by using a Quant-iT Protein Assay Kit (Invitrogen, Waltham, MA, USA).

### 2.5. Western Blot Protocol

Western blotting was performed according to a standard protocol [34,35]. Equal amounts of protein (40 μg/well) were subjected to SDS-PAGE (12% gels, Bio-Rad, Hercules, CA, USA) and transferred to nitrocellulose membranes. After blocking in TBST (Tris-buffered saline, 0.1% Tween 20) with 5% BSA (Bovine Serum Albumin, Invitrogen, Waltham, MA, USA), membranes were probed overnight at 4 °C with the corresponding primary antibodies, e.g., anti-IL-1β (1:500; Abcam, Cambridge, UK); anti-IL-6 (1:750; Novus Biologicals, Centennial, CO, USA); anti-TNF-α, (1:1000; Invitrogen, Waltham, MA, USA); anti-NF-κB, (1:1000; Invitrogen, Waltham, MA, USA). Anti-actin antibody (1:1000, Santa Cruz, CA, USA) was used for the loading control. After the washing procedure, membranes were incubated with alkaline phosphatase-conjugated secondary antibodies, IgG (1:5000 Santa Cruz, CA, USA) for 1 h at room temperature. The immunoreactive bands were visualized by a colorimetric detection kit (NBT-BCIP; ThermoFisher, Waltham, MA, USA) and protein amounts were analyzed with the ImageJ program (1.46r, NIH, Bethesda, MD, USA).

### 2.6. Real Time PCR (Quantitative-q-PCR)

For quantification of mRNA expression in tissues total RNAs were isolated by using the RNAzol RT solution (MRC, Cincinnati, OH, USA) according to the manufacturer’s instructions. After completion of RNA isolation, RNA concentration and purity were calculated with NanoDrop 2000 (Thermo Scientific, Waltham, MA, USA). For this purpose, 1 µL RNA samples were pipetted in the device for the determination of 260/280 and 260/230 ratios. Concentrations of all RNA samples were equalized before reverse transcription. RNAs were reverse transcribed into cDNA by using the Script cDNA Synthesis Kit (Jena Bioscience, Jena, Germany). The resulting cDNA was amplified by qRT-PCR by using qPCR GreenMaster with the UNG Kit (Jena Bioscience, Jena, Germany). The real-time conditions were carried out on the CFX-96 Real-Time PCR System (Bio-Rad, Hercules, CA, USA) as follows: 50 °C, 2 min; 95 °C, 2 min; followed by 35 cycles of 95 °C, 15 s; 56 °C, 20 s; and 72 °C, 30 s. Relative mRNA transcripts levels were calculated according to the delta CT method (2^−∆∆CT^) and the relative expression of each gene was normalized to that of HPRT. Primers were obtained from LGC Biosearch Technologies (Novato, CA, USA) (Table 1). All measurements were performed in triplicate and the specificity of amplicons was verified by melting curve analysis. The specific primers used are shown in Table 1.

### 2.7. Statistical Analyses

Statistical analyses were performed using GraphPad Prism (GraphPad Software, La Jolla, CA, USA). After checking for normal distribution, an analysis of variance (ANOVA) test using the post hoc Tukey test was applied. Results were expressed as the mean ± SD and a *p*-value of <0.05 was considered significant.

## 3. Results

### 3.1. Blood Glucose Levels and Biochemical Parameters

Blood glucose levels in diabetes-induced groups were found to be significantly (*p* < 0.001) higher, both before the start of melatonin administration and after the finalization of the melatonin administration process at the end of 6 weeks. Melatonin-administered groups (Groups 3 and 4) showed slightly lower blood glucose levels compared to the DM group. However, the difference was not significant (*p* > 0.05) (Table 2).

AST, ALT, and BUN levels were significantly higher in the plasma of diabetic rats as compared to the levels of the control group (Table 3). However, the administration of melatonin to diabetic rats was associated with significantly lower levels of AST, ALT (both *p* < 0.05 compared to diabetic rats), and BUN (*p* < 0.01). The MT2 receptor antagonist luzindole did not attenuate the beneficial effects of melatonin regarding these parameters (*p* > 0.05).

Triglycerides, VLDL, and cholesterol levels were significantly higher in the DM group and melatonin treatment led to significantly lower levels of these parameters in diabetic rats (Table 3). The addition of luzindole to melatonin-treated diabetic rats affected the results on blood lipid levels, assuming that the MT2 receptor might be mechanistically involved in the effects of melatonin on these parameters.

### 3.2. Q-PCR

**Figure 1:**
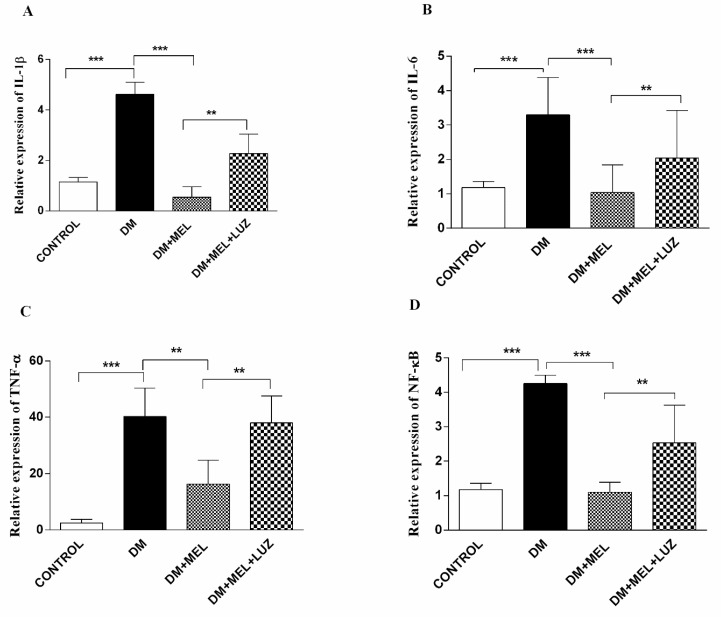
Q-PCR relative expression of cytokine levels in the liver. IL-1β (**A**), IL-6 (**B**), TNF-α (**C**), NF-κB (**D**) levels in liver tissues of control, DM, DM plus treatment with melatonin (500 µg/kg/day), and DM plus treatment with luzindole (0.25 g/kg/day) and melatonin (500 µg/kg/day). Values are represented as the mean ± SD. (**A**) There is a significant increase in the DM group compared to the control group (*p* < 0.001) and relative expression of IL-1β decreased significantly in DM + MEL group compared to the DM group. There is a significant increase in the DM + MEL + LUZ group compared to the DM + MEL group (*p* < 0.01). (**B**) There is a significant increase in the DM group compared to the control group (*p* < 0.001). In the DM + MEL group relative expression of IL-6 deceased significantly compared to the DM group *(p* < 0.001) and there is a significant increase in the DM + MEL + LUZ group compared to the DM + MEL group (*p* < 0.01). (**C**) There is a significant increase in the DM group compared to the control group (*p* < 0.001) and a significant decrease in TNF-α expression in DM + MEL group compared to the DM group (*p* < 0.01). In DM + MEL + LUZ group relative expression of TNF-α increased significantly compared to the DM + MEL group (*p* < 0.01). (**D**) There is a significant increase in NF-κB expression levels in DM group compared to the control group (*p* < 0.001); relative expression of NF-κB decreased in DM + MEL group compared to the DM group (*p* < 0.001).There is a significant increase in NF-κB expression levels in DM + MEL + LUZ group compared to the DM + MEL group (*p* < 0.01). ** = *p* < 0.01, *** = *p* < 0.001.

Gene expression levels of the inflammatory cytokines IL-1β, IL-6, TNF-α, and NF-κB were analyzed in liver-, adipose-, and brain tissues. In the liver tissue, relative expression of all four inflammatory parameters increased dramatically in the DM group with the melatonin treated DM group showing significant lower expression of all four inflammatory markers (IL-1β, IL-6, NF-κB = *p* < 0.001 compared to the diabetic group; TNF-α = *p* < 0.01) (Figure 1). The addition of luzindole to the MEL + DM group partly abolished the positive effects of melatonin on all cytokine levels (*p* < 0.01, Figure 1).

**Figure 2:**
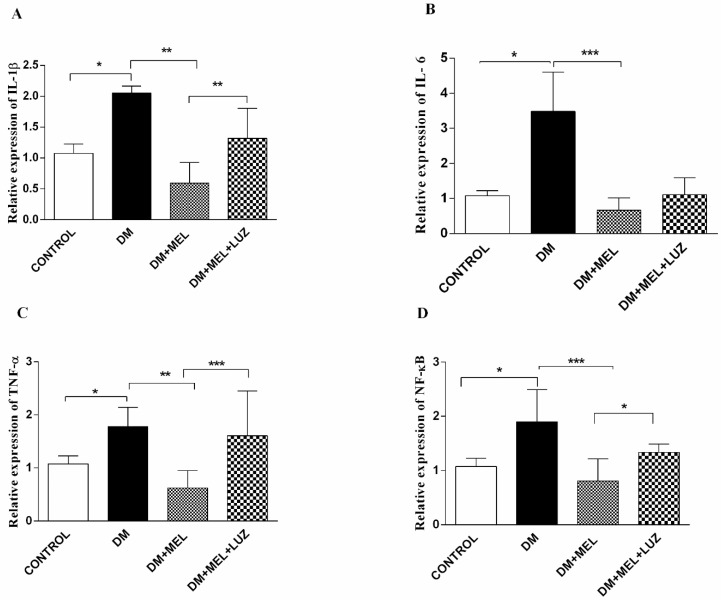
Q-PCR relative expression of cytokine levels in adipose tissue. IL-1β (**A**), IL-6 (**B**), TNF-α (**C**), NF-κB (**D**) levels in adipose tissues of control, DM, DM plus treatment with melatonin (500 µg/kg/day), and DM plus treatment with luzindole (0.25 g/kg/day) and melatonin (500 µg/kg/day). Values are represented as the mean ± SD. (**A**) There is a significant increase in the DM group compared to the control group (*p* < 0.05). Relative expression of IL-1β decreased significantly in DM + MEL group compared to the DM group (*p* < 0.01). In the DM + MEL + LUZ group, expression of IL-1β increased significantly compared to the DM + MEL group (*p* < 0.01). (**B**) There is a significant increase in IL-6 gene expression levels in DM group compared to the control group (*p* < 0.05). In DM + MEL group, relative expression of IL-6 decreased compared to the DM group (*p* < 0.001). (**C**) There is a significant increase in TNF-α levels in the DM group compared to the control group (*p* < 0.05). In DM + MEL group, TNF-α expression levels decreased compared to the DM group (*p* < 0.01). There is a significant increase in the DM + MEL + LUZ group compared to the DM + MEL group (*p* < 0.001). (**D**) There is a significant increase in NF-κB expression levels in DM group compared to the control group (*p* < 0.05). In DM + MEL group NF-κB expression levels decreased significantly compared to the DM group (*p* < 0.001). In DM + MEL + LUZ group, NF-κB levels increased compared to the DM + MEL group (*p* < 0.05). Significant differences = *. * = *p* < 0.05, ** = *p* < 0.01, *** = *p* < 0.001.

In the adipose tissue, all cytokine gene expression levels were significantly higher in the DM group compared to control rats (*p* < 0.05) and melatonin treatment in DM rats resulted in significantly lower cytokine gene expression levels compared to the untreated DM group (Figure 2). Luzindole treatment increased IL-1β, TNF-α, and NF-κB gene expression levels significantly compared to MEL-treated DM groups (*p* < 0.01, *p* < 0.001, *p* < 0.05, respectively) (Figure 2).

**Figure 3:**
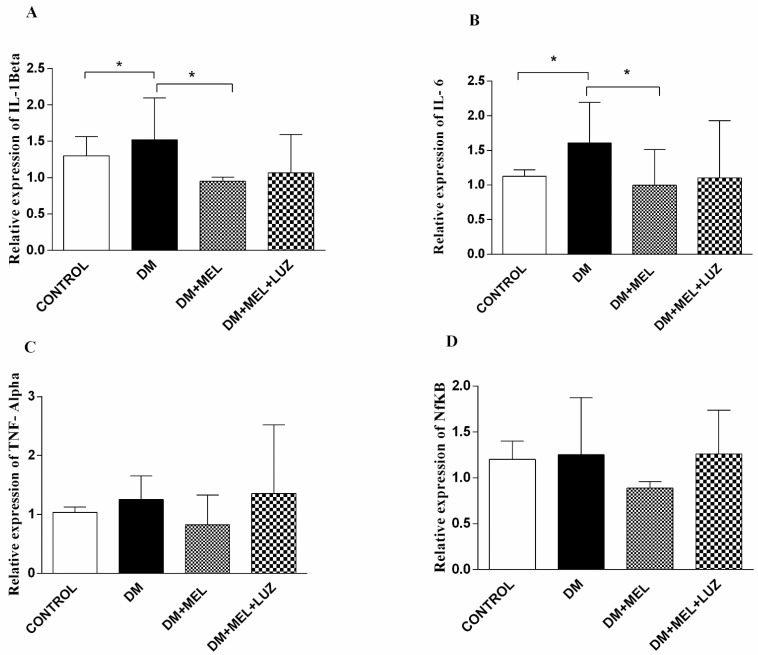
Q-PCR relative expression of cytokine levels in brain tissue. IL-1β (**A**), IL-6 (**B**), TNF-α (**C**), NF-κB (**D**) levels in brain tissues of control, DM, DM plus treatment with melatonin (500 µg/kg/day), and DM plus treatment with luzindole (0.25 g/kg/day) and melatonin (500 µg/kg/day). Values are represented as the mean ± SD. (**A**) In the DM group relative expression of IL-1β increased significantly compared to the control group (*p* < 0.05). In DM + MEL group, IL-1β expression decreased compared to the DM group (*p* < 0.05). (**B**) There is a significant increase in the relative expression of IL-6 levels in the DM group compared to the control group (*p* < 0.05). Expression levels decreased in DM + MEL group according to the DM group (*p* < 0.05). Significant different = *. * = *p* < 0.05. There was no significant difference between the groups for the (**C**,**D**). (**C**,**D**)There was no significant difference between the groups.

In the brain tissue of DM rats, IL-1β and IL-6 cytokine levels were significantly higher compared to controls (*p* < 0.05) with melatonin-treated diabetic rats showing lower values (*p* < 0.05) of these two cytokines (Figure 3). No significant intergroup differences between DM and DM + MEL were observed in the expression levels of the other two cytokines genes Furthermore, luzindole treatment did not affect the beneficial effects of melatonin regarding IL-1β and IL-6 (Figure 3).

### 3.3. Western Blot

IL-1β, IL-6, TNF-α, and NF-κB protein levels were studied by Western blotting in the liver tissue. β-actin was used as the reference protein and the cytokine band absorbance level to β-actin’s level in each well was compared. Densitometric analysis were performed by the ImageJ program (1.46r, NIH, Bethesda, MD, USA)

**Figure 4:**
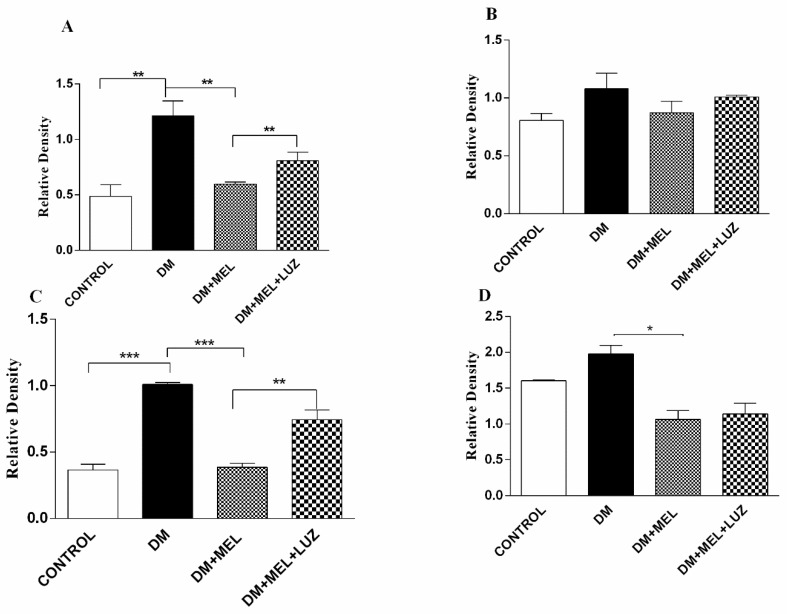
Western blot results of cytokine levels in liver tissue. IL-1β (**A**), IL-6 (**B**), TNF-α (**C**), NF-κB (**D**) relative density in liver tissues of control, DM, DM plus treatment with melatonin (500 µg/kg/day), and DM plus treatment with luzindole (0.25 g/kg/day) and melatonin (500 µg/kg/day). Values are represented as the mean ± SD. Significant different = *. * = *p* < 0.05, ** *= p* < 0.01, *** = p < 0.001.

In the liver tissue of DM rats, IL-1β and TNF-α protein levels were significantly higher than in the liver of the control animals (Figure 4). The addition of melatonin to diabetic rats led to significantly lower levels of IL-1β (*p* < 0.01), TNF-α (*p* < 0.001) and NF-κB (*p* < 0.05). The MT2 receptor appeared to be at least partly involved in the effects of melatonin, since protein levels of IL-1β, and TNF-α were significantly higher in the DM + MEL + LUZ group compared to the DM + MEL group. IL-6 levels were not significantly different between the groups (Figure 4).

### 3.4. Immunohistochemistry

**Figure 5:**
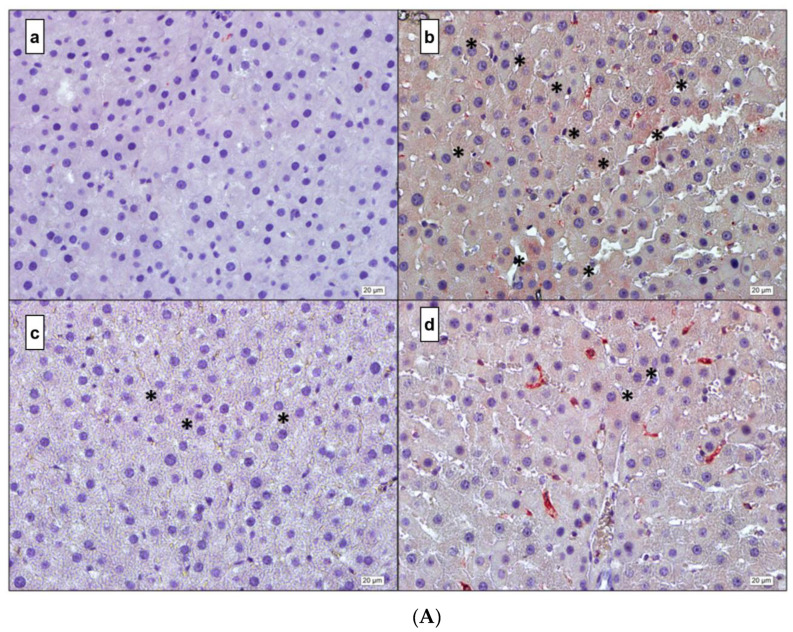

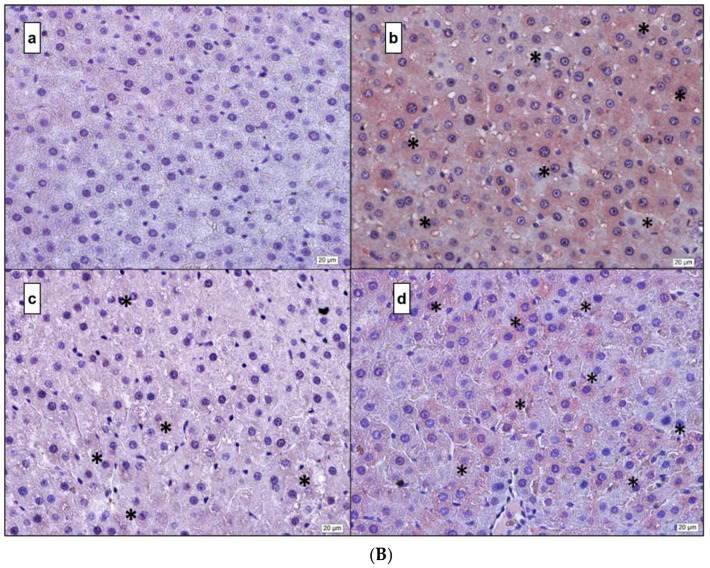

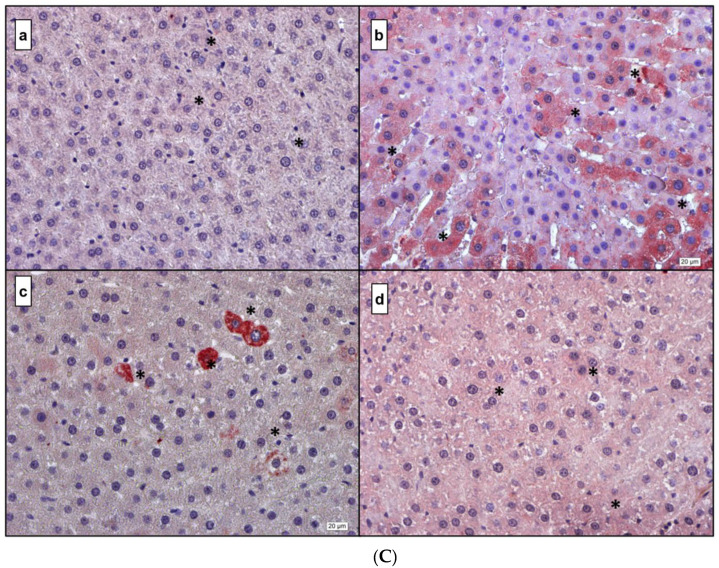

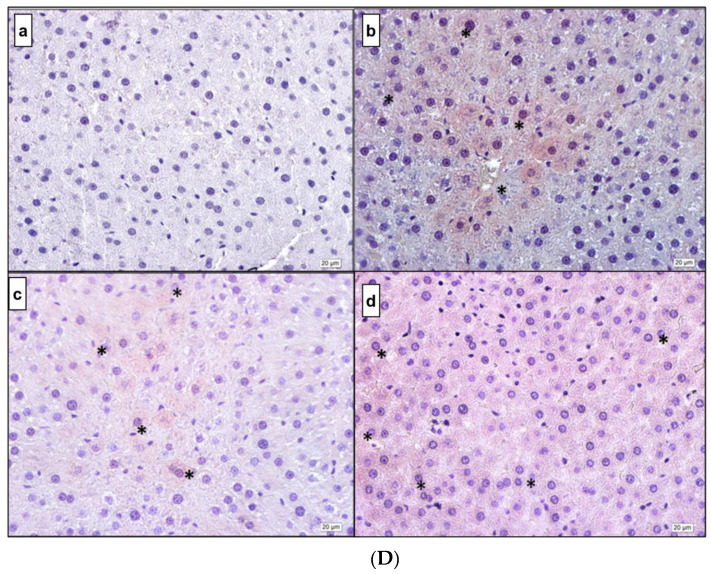
(**A**) IL-1β. (**a**) Control; (**b**) DM; (**c**) DM + MEL; (**d**) DM + MEL + LUZ. (**B**) IL-6. (**a**) Control; (**b**) DM; (**c**) DM + MEL; (**d**) DM + MEL + LUZ. (**C**) TNF-α. (**a**) Control; (**b**) DM; (**c**) DM + MEL; (**d**) DM + MEL + LUZ. (**D**) NF-κB. (**a**) Control; (**b**) DM; (**c**) DM + MEL; (**d**) DM + MEL + LUZ. * represents the positive reaction.

**Figure 6:**
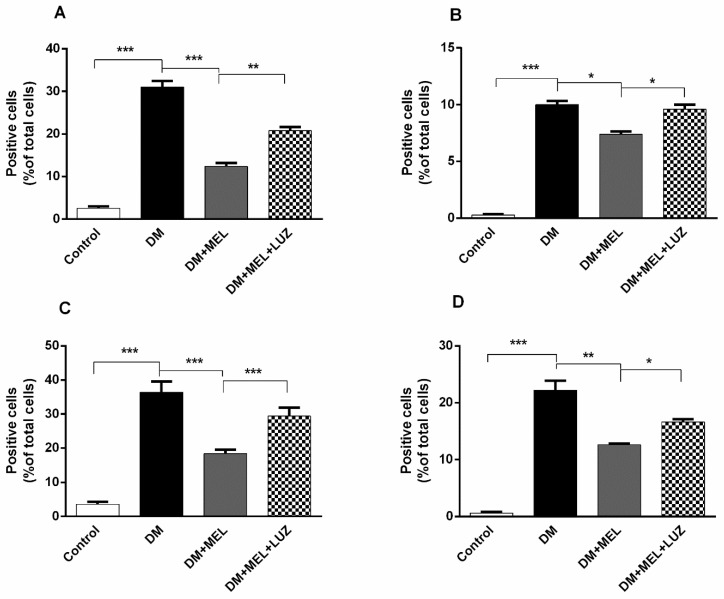
(**A**) Mean ratio of number of IL-1β-positive cells to the total number of cells in liver tissue. Ratio of IL-1β-positive cells to total number of cells was found to be increased significantly in DM group compared with control group (*p* < 0.001). Melatonin administration reduced the ratio dramatically (*p* < 0.001). Luzindole treatment abolished the positive effects of melatonin in a significant manner (*p* < 0.01). (**B**). Mean ratio of number of IL-6-positive cells to the total number cells in liver tissue. In DM group, ratio of IL-6-positive cells to total number of total cells was found to be increased significantly compared to the control group (*p* < 0.001). Ratio was declined in MEL group when compared to DM group (*p* < 0.05). Luzindole administration increased the number of positive cells when compared to MEL group (*p* < 0.05). (**C**). Mean ratio of number of TNF-alpha positive cells to the total number of cells in liver tissue. In DM group ratio of TNF-alpha positive cells to total number of cells was elevated significantly (*p* < 0.001). Melatonin administration decreased the ratio dramatically (*p* < 0.001). Luzindole administration dramatically increased the number of positive cells when compared to MEL group (*p* < 0.001). (**D**). Mean ratio of number of NF-κB positive cells to the total number of cells in liver tissue. In DM group ratio of NF-κB positive cells to the total number of cells was elevated significantly (*p* < 0.001). Melatonin administration decreased the number of positive cells (*p* < 0.01). Luzindole administration increased the ratio of positive cells in diabetic rats when compared to MEL group (*p* < 0.05). Significant different = *, * = *p* < 0.05, ** = *p* < 0.01, *** = *p* < 0.001.

Immunostainings of all cytokine levels (IL-1β, IL-6, TNF-α, and NF-κB) exhibited an intense immunoreactivity in the DM group. A representative immunohistochemistry pictures and quantification of immunostaining from all cytokine levels in the liver is presented in Figure 5 and Figure 6. A low level of cytokine immunoreactivity is observed in the DM + MEL group. The DM + MEL + LUZ group exhibits a moderate intensity of cytokine immunoreactivity.

Figure 5A representative microscopic pictures of IL-1β immunoreactivity of liver cross-sections in all experimental groups. (a) Control group: a weak immunoreactivity can be observed. (b) DM group: the immunoreactivity pattern is strong and diffused in the cytoplasm of the hepatocytes. (c) DM + MEL group: immunoreactivity is observed in the cytoplasm of a few hepatocytes. (d) Luz group: stronger immunoreactivity is observed compared to the DM + MEL group.

Figure 5B representative microscopic pictures of IL-6 immunoreactivity of liver cross-sections of all experimental groups. (a) Control group: IL-6 immunoreactivity in the cytoplasm of hepatocytes is negative. (b) DM group: IL-6 immunoreactivity can be seen widely at the cytoplasmic level in hepatocytes. (c) DM + MEL group: IL-6 immunoreactivity in hepatocytes is rarely observed. (d) Luzindole group: IL-6 immunoreactivity is considerably higher than the DM + MEL group.

Figure 5C representative microscopic pictures in TNF-α immunoreactivity of liver sections in all experimental groups. (a) Control group: a weak TNF-α immunoreactivity can be seen in the hepatocytes. (b) DM group: a considerably higher immunoreactivity can be seen along hepatic plates compared to the control group. (c) DM + MEL group: TNF-α immunoreactivity is observed at a moderate level, the severity of immunoreaction is reduced compared to the DM group. (d) Luzindole group: the immunoreactivity is considerably higher than the DM + MEL group.

Figure 5D representative microscopic pictures of NF-κB immunoreactivity of cross-sections from the liver in all experimental groups. (a) Control group: negative immunoreactivity in the cytoplasm of hepatocytes is observed (b) DM group immunoreactivity of NF-κB is higher than the control group. (c) DM + MEL group: relatively weak immunoreactivity is observed compared to the DM group. (d) Luzindole group: the immunoreactivity is considerably higher than the DM + MEL group.

## 4. Discussion

This study in particular showed that administration of melatonin significantly reduced inflammation in the liver and adipose tissue of diabetic rats with the positive effects being partly explained by the involvement of the MT2 receptor. In addition, some metabolic improvements were detected in melatonin-treated diabetic animals.

Melatonin has been related to glucose and insulin homeostasis regulation in different ways. For example, pinealectomized rats showed insulin resistance and glucose intolerance [36], and in Goto Kakizaki rats, a model for type 2 diabetes, nocturnal melatonin secretion was reduced whereas higher than normal levels of insulin were reported [37,38]. Higher levels of insulin are considered a hallmark of T2D [38]. In addition, in T1DM melatonin levels were significantly lower when compared to the control group [39].

In the present work, there was no obvious difference in the blood glucose levels between diabetic and melatonin administrated in diabetic groups. Although there are studies showing an effect of melatonin on blood glucose levels in rats with diabetes [40,41], there are also some similar studies in the scientific literature where melatonin did not alter the glucose levels in diabetes mellitus [42,43,44]. The marginal effects of melatonin on blood glucose levels in our study could be related to the melatonin dosage used.

It is well known that inflammatory markers are elevated in insulin-resistant T2DM patients [45,46,47] with an impaired balance between T cell subtypes might playing an important role in the deterioration of glucose homeostasis in both type 1 and type 2 diabetes [48]. Moreover, experimental studies in recent years have shown that interferon-γ expression is increased in diabetic mice and induces adipose tissue inflammation [49]. Furthermore, c-Jun N- kinases (JNKs), which are key regulators of inflammation, exhibit a marked increase in obesity. In this regard, an increased expression of TNF-α, which is a potent regulator of JNKs, has been demonstrated in obese mice connecting a link between obesity and insulin resistance [50]. The JNK pathway is activated in several tissues in DM and inhibition of JNK is known to ameliorate insulin resistance [51]. In addition, JNK knockdown mice have a decreased expression of pro-inflammatory cytokines such as TNF-α or IL-6, which might protect against insulin resistance in T2DM [52].

Overall, the role of inflammation in DM has been widely investigated [53,54]. On the other hand, there are only scarce data available regarding the relationship between melatonin, inflammation, and type 2 diabetes and its complications. For example, in previous studies with young ZDF (Zucker diabetic fatty) rats, an experimental model of the metabolic syndrome and type 2 diabetes, oral administration of melatonin reduced the levels of pro-inflammatory cytokines such as IL-6, TNF-α, and CRP, oxidative stress or low-grade inflammation [55]. Furthermore, Ozkanlar et al. reported that melatonin administration decreases IL-1β levels in the serum of rats with induced T1DM [56]. Moreover, in T2DM patients with chronic periodontitis, melatonin supplementation decreased the levels of IL-6 in serum [57]. In addition, melatonin was shown to prevent the production of pro-inflammatory cytokines such as IL-1β and TNF-α in diabetic retinopathy [58].

Melatonin significantly reduced NF-κB levels in our diabetic rats. NF-κB is a transcription factor, which mediates the production of the pro-inflammatory cytokines TNF-α, IL-1β, and IL-6, and also plays an important role in innate immunity. Previous studies have indicated that melatonin inhibits the transcriptional activation of TNF-α and IL-1β [59,60] by blocking NF-κB binding to DNA [61]. Moreover, in ovarium tissue sections in ovarium injured diabetic rats [62] and in experimental diabetic neuropathy [63], NF-κB immunoexpression was found to be significantly lower in melatonin-treated diabetic rats.

Melatonin primarily exerts its effects through two G-protein coupled membrane receptors (GPCRs), MT1 and MT2, respectively [24]. Although receptor distributions may vary, melatonin receptors are expressed in many different organs and tissues with the MT2 receptor also being found in the endocrine pancreas [64]. A study by Ramracheya et al. suggested that the stimulation of insulin secretion is either through melatonin’s interaction with beta-cell MT2 receptors or an indirect stimulatory effect [65]. In another study assessing the impact of the human recombinant MT2 receptor on insulin secretion, the authors indicated that functional hMT2expression led to a reduction in insulin release [66]. MT receptors have also been identified in leukocytes [67,68,69] and melatonin is suggested to modulate (or regulate) the immune system by, for example, stimulating lymphocytes via these receptors, predominantly via the MT2 receptor [70]. Complete removal of the pineal gland causes partial deterioration in the immune responses of rats [71,72], and administration of melatonin in pinealectomized animals reverses the negative effect on immune responses [72].

Luzindole is a selective melatonin receptor antagonist and has a high affinity for the MT2 receptor. It was, for example, observed that mice treated with luzindole produced lower levels of IgG than non-treated controls [70]. In our study, we also aimed to investigate whether melatonin exerted its possible anti-inflammatory effects via the MT2 receptor. We demonstrated that some of the potent anti-inflammatory effects of melatonin were lost, at least tendentially, after the application of luzindole. In accordance, for example, in mesenchymal stem cells exposed to inflammation, it was shown that melatonin reduced the amount of reactive oxygen species in these cells and acted as an anti-inflammatory agent; however, administration of luzindole reversed this effect [73]. In another study, it was shown that melatonin reduces colonic and gastric inflammation, but the administration of luzindole abolished these beneficial effects of melatonin [74]. All these data, together with ours, demonstrate that MT2 receptors have a role in the anti-inflammatory effects of melatonin. In contrast to the effects on inflammatory markers, blocking the melatonin MT2 receptor in our study did not reverse the effects on metabolic parameters, such as blood glucose, AST, ALT, and BUN levels. The reason could be a disconnection between the anti-inflammatory and systemic effects of melatonin. Although the results of our study are in line with other studies examining the effects of melatonin and luzindole on inflammation, it is probably the first extensive study on diabetes, inflammation, and melatonin in the literature.

## 5. Conclusions, Strengths and Limitations

Overall, our findings are compatible with the available data of former studies suggesting that the administration of melatonin reduces inflammation in diabetes mellitus. The strengths of our study are the application of a variety of methods, including different tissues with four different inflammation markers, and also studying the potential involvement of the MT2 receptor in mediating the potential beneficial effects of melatonin. Nevertheless, our study has also limitations. We did not study the involvement of the MT1 receptor. Nevertheless, recent data indicated that MT2 receptors have a more direct involvement in T2DM [75]. Besides, we used relatively low doses of melatonin compared to previous investigations [42,76]. Furthermore, we did not determine blood endogenous melatonin levels before the administration of the exogenous melatonin. Moreover, we did not address oxidative stress markers in our study. Oxidative stress has also been suggested to be involved in inflammation [77]. However, analyzing oxidative stress was beyond the scope of our study and could be investigated in the future.

## 6. Future Perspectives

Future clinical studies could address the effect of melatonin supplementation in patients with diabetes mellitus, preferably with a higher grade of inflammation. It would also be interesting to include older diabetic patients since it is known that aging is associated with a lower nocturnal melatonin secretion [78,79], which could aggravate the inflammatory state. Additionally, aging is associated with a chronic low-grade pro-inflammatory state [80] suggesting a multiplying effect of low melatonin, diabetes, and inflammation.

## Figures and Tables

**Table 1:** Primers used in real-time polymerase chain reaction analysis.

Primer Name	Forward Primer Sequence (5′-3′)	Reverse Primer Sequence (3′-5′)
TNF-a (NM_012675.3)	GCAGATGGGCTGTACCTTATC	GAAATGGCAAATCGGCTGAC
IL-6 (NM_012589.2)	GTCTTCTGGAGTTCCGTTTCT	GGGTTTCAGTATTGCTCTGAATG
IL-1B (NM_031512.2)	CGTGGGATGATGACGACCTG	TGGGTGTGCCGTCTTTCATC
NF-kB (NM_001276711.1)	GGTTACGGGAGATGTGAAGATG	GTGGATGATGGCTAAGTGTAGG
HPRT (Housekeeping) (NM_012583.2)	GACCTCTCGAAGTGTTGGATAC	TCAAATCCCTGAAGTGCTCAT

HPRT: hypoxanthine phosphoribosyltransferase.

**Table 2:** Blood glucose levels (mg/dL) in experimental groups.

Blood Glucose Levels (mg/dL)	Control		DM		DM + MEL		DM + MEL + LUZ	
	B	A	B	A	B	A	B	A
	92 ± 11	94 ± 9	423 ± 22 ***	415 ± 28 ***	416 ± 25 ***	390 ± 24 ***	421 ± 32 ***	405 ± 20 ***

B = before the start of melatonin administration. A = at the end of the 6-week period. Blood glucose levels of control and experimental groups before the start of melatonin administration and after the finalization of melatonin administration process at the end of 6 weeks. The DM group received only vehicle. DM and melatonin groups had significantly higher blood glucose levels than the control group (*p* < 0.001). Significant different *** = *p* < 0.001 compared to control.

**Table 3:** Levels of biochemical parameters in experimental groups.

	CONTROL	DM	DM + MEL	DM + MEL + LUZ
AST (U/L)	127.0 ± 24.31	232.7 ± 65.84 *	146.9 ± 17.42 ^§^	168.7 ± 16.25
ALT (U/L)	65.04 ± 10.98	120.1 ± 25.45 ***	83.82 ± 13.61 ^§^	80.60 ± 23.93
BUN (mg/dL)	13.89 ± 1.65	29.08 ± 4.95 ***	23.09 ± 1.15 ^§§^	21.15 ± 5.69
TRIGLYCERIDE (mg/dL)	104.9 ± 26.13	247.2 ± 44.99 **	163.4 ± 21.58 ^§^	203.8 ± 42.73 ^#^
VLDL (mg/dL)	23.13 ± 6.7	68.00 ± 28.69 **	31.17 ± 5.269 ^§^	42.80 ± 12.26 ^#^
CHOLESTEROL (mg/dL)	49.70 ± 5.35	69.74 ± 8.71 ***	51.06 ± 1.24 ^§§^	57.33 ± 4.8 ^#^

Results of biochemical analyses in the different groups. Values are represented as the mean ± SD. Significant different compared to controls = *. Significant different compared to DM = ^§^. Significant different compared to MEL = ^#^. *, ^§^, ^#^ = *p* < 0.05; **, ^§§^ = *p* < 0.01, *** = *p* < 0.001.

## Data Availability

Data can be obtained by request from the corresponding author.
